# Biodiversity of soil algae in the farmlands of mid-Taiwan

**DOI:** 10.1186/1999-3110-54-41

**Published:** 2013-09-27

**Authors:** Ching-Su Lin, Tsuan-Ling Chou, Jiunn-Tzong Wu

**Affiliations:** 1 Institute of Ecology and Evolutionary Biology, National Taiwan University, 1, Roosevelt Road Section 4, Taipei, 106 Taiwan; 2 Biodiversity Research Center, Academia Sinica, 128, Academia Road Section 2, Nankang, Taipei, 115 Taiwan

**Keywords:** Biodiversity, Farmland, Soil algae, Soil cyanobacteria, Subtropical Taiwan

## Abstract

**Background:**

Very little information is available about soil algae in Taiwan. In this study, we investigated for the first time the soil algae inhabiting five types of farmland in mid-Taiwan: rice paddies, vegetable farms, tea plantations, sugar cane farms, and orchards.

**Results:**

Sixty-four taxa belonging to 33 genera of cyanobacteria, diatoms, green algae, and euglenoids were identified on the basis of fine structures observed under optical and electron microscopes and rDNA sequencing. The majority of the isolates were of the genera *Oscillatoria*, *Navicula*, *Nitzschia*, and *Pinnularia*. Five species were reported for the first time in Taiwan, namely *Microcoleus paludosus*, *M. subtorulosus*, *Navicula subminuscula*, *Nitzschia levidensis*, and *Ni. pusilla*.

**Conclusions:**

The distribution and diversity of these species was shown to be strongly dependent on habitat, with the highest diversity of green algae in the orchards, a fairly high diversity of diatoms and cyanobacteria in the rice paddies, and a relatively low diversity in the tea plantations and sugar cane farms. The humidity and acidity were the most important environmental factors influencing the diversity of soil algae in these farmlands.

**Electronic supplementary material:**

The online version of this article (doi:10.1186/1999-3110-54-41) contains supplementary material, which is available to authorized users.

## Background

Soil is the most important non-aqueous habitat for algae and cyanobacteria (blue-green algae) (Zenova et al., 1995). Due to their high capacity for morphological and physiological adaptations to different environments, both algae and cyanobacteria often act as pioneer micro-organisms in terrestrial ecosystems (Metting, 1981; Hoffmann, 1989). In farmlands, soil algae and cyanobacteria play an important role because they both have tremendous potential to serve as sources of nitrogen and carbon for other organisms. Soil fertility is generally improved by the organic matter produced by these organisms (Mishra and Pabbi, 2004). Soil algae excrete growth-promoting substances such as hormones, vitamins, amino acids, and organic acids that affect other organisms in many ways (Roger and Reynaud, 1982; Wilson, 2006). The presence of soil algae also stabilizes the soil surface and thus reduces erosion (Evans and Johansen, 1999; Hu et al., 2004). The polysaccharides produced by some soil algae increase soil porosity, aggregation, and water-holding capacity (Roger and Reynaud, 1982; Choudhary et al.*,*^2007^). Soil algae are also ameliorators in the reclamation of saline and metals, thereby improving soil quality (Rai et al., 1998; Whitton and Potts, 2000).

Several studies have noted that the inoculation of farm soils with algae increases grain yields by 15-25% (Yanni et al., 1992; Gurung and Prasad, 2005; Song et al., 2005). Cyanobacteria are the preferred bio-fertilizer, because they perform two key biological processes: oxygenic photosynthesis and nitrogen fixation (Relwani, 1963; Ernst et al., 1992). The commonly used nitrogen-fixing species are of genera *Anabaena*, *Calothrix*, *Nostoc*, *Schizothrix,* and *Scytonema* (Kannaiyan, 1990; Rai et al., 2000).

The identification of unicellular or crustose algae is problematic, especially when only limited sample material is available (Burja et al., 2001; Presting, 2006). In recent years, molecular tools have improved the precision of species identification and allowed eco-physiological and biochemical approaches to biodiversity assessment (Srivastava et al., 2007). Although some soil algae have been studied in certain localities in various regions across the world, information about the diversity and ecological characteristics of soil algae in Taiwan is very limited. In this study, we conducted a survey of soil algal diversity in different types of farmland in mid-Taiwan using both morphological observations with optical and scanning electron microscopes and molecular data from rDNA sequences.

## Methods

### Sampling and morphological observations

Soil samples from different types of farmland in the counties of Miaoli, Taichung, Changhua and Nantou from mid-Taiwan, including rice (*Oryza sativa* Linn.) paddies, vegetable farms (mainly growing white cabbage, *Brassica oleracea* var. *capitata* L.), tea (*Camellia sinensis* L.), and sugar cane (*Saccharum officinarum* L.) plantations, and orchard farms (mainly growing orange *Citrus sinensis* L., and tangerine *C. reticulata* Blanco.) were collected seasonally over the time from May 2010 to May 2011, namely May, August, and November of 2010 and February, May of 2011, by removing the surface debris from five to seven randomly selected sites and scraping about 500 g of soil from the upper centimeter of soil at each site. After thorough mixing and sieving (through a 1.0-mm mesh), the soil samples were stored in polythene bags and brought to the laboratory. About 10 g of each sample was placed in a flask and diluted 100-fold with distilled water. After shaking for 1 h, the soil suspensions were fixed with formaldehyde solution (at a final concentration of 1%). A part of each fixed soil suspension was boiled in 10% H_2_O_2_ solution to remove any organic material and was repeatedly rinsed with distilled water to obtain cleaned diatom frustules (Fujita and Ohtsuka, 2005). Algae were identified by direct examination using an AxioStar microscope (Zeiss, Germany) equipped with transmitted light, phase contrast, and differential interference contrast (DIC, Nomarski) illumination. The findings were photo-documented using a ProgRes digital microscope camera (Jena, Germany).

### Scanning electron microscope (SEM)

To observe the fine structures of the diatom frustules, about 1 g of each sample was treated with sulfuric/acetic (1: 9) acid solution, as described by Chen and Wu (1999). The samples were then dehydrated through an alcohol series and dried with a critical point dryer (Hitachi HCP-2). The dried diatoms were mounted on an aluminum stub and coated with gold by a sputter coater (Edwards S150A) and viewed on an FEI Quanta 200 scanning electron microscope (SEM).

The determination and nomenclature of the organisms were carried out with reference to the relevant texts (Desikachary, 1959; Prescott, 1962; Patrick and Reimer, 1975; Anagnostidis and Komárek, 1990; Komárek and Anagnostidis, 1999, 2005; John et al., 2002; Kobayasi et al., 2006).

### Isolation, culture, and DNA sequencing

A total of 0.1 mL of the soil suspensions without formaldehyde solution was inoculated in triplicate on both liquid and solid (1.5% agar) mediums of BG11 (Stanier et al., 1971) and NC (Kuhl, 1962), respectively. The inoculations were kept in a greenhouse under a 12:12 light-dark (LD) cycle and illuminated with cool white fluorescent light at 40 μE m^−2^ s^−1^ and 25°C. The isolates were microscopically observed and prepared for subsequent DNA isolation and further analysis.

The DNA of the soil algae was extracted using the phenol-chloroform protocol (Saunders, 1993). Amplification was carried out using the PCR primer pair 359 F (5′ GAA TYT TCC GCA ATG GGC 3′) and CYA781R (an equimolar mixture of CYA781R-a (5′GAC TAC TGG GGT ATC TAA TCC CAT T3′) and CYA781R-b (5′ GAC TAC AGG GGT ATC TAA TCC CTT T 3′)) of 16S rDNA for the cyanobacteria (Nübel et al., 1997); primer pair P73F (5′AAT CAG TTA TAG TTT ATT TGR TGG TAC C3′) and p47R (5′TCT CAG GCT CCC TCT CCG GA3′) of 18S rDNA for the eukaryotic algae (Bérard et al., 2005); or primer pair p23SrV_f1 (5′ GGA CAG AAA GAC CCT ATG AA 3′) and p23SrV_r1 (5′ TCA GCC TGT TAT CCC TAG AG 3′) of 23S rRNA for both (Sherwood and Presting, 2007). The PCR amplification was performed in an Eppendorf Mastercycler gradient S thermal cycler (Eppendorf, Hamburg, Germany) using a 50 μL solution mixture consisting of 5.0 μL of 10X PCR buffer, 4.0 μL of 2.0 mM MgCl_2_ (Promega), 3.0 μL of 1.0% BSA solution, 2.0 μL of each primer (0.4 mM), 2.0 μL (20 mM) of each dNTP, 0.2 μL of *Taq* polymerase (Promega), 26.0 μL of H_2_O (bidist.), and 1.0 μL of genomic DNA. The cycling conditions consisted of heating at 94°C for 5 minutes, followed by 35 cycles of 94°C for 1 min, 60°C for 1 min for the 16S and 18S rDNA primers (55°C for the 23S primers), 72°C for 1 min, and a final extension time of 72°C for 10 minutes. The PCR products were visualized on 1% agarose gel stained with ethidium bromide (EtBr), further purified using a Qiagen PCR purification kit (Stratagene, CA, USA).

The PCR products were sequenced commercially in both directions. The forward and reverse-complementary of the reverse sequences obtained were aligned to check the quality of the sequences. Ambiguous bases were checked and altered using the BioEdit program. Sequences were compared to known samples and environmental samples using the BLAST search tool on the National Center for Biotechnology Information (NCBI) website (http://www.ncbi.nlm.nih.gov).

### Physicochemical properties of soil

Soil samples were soaked in deionized water (1: 5 v/v) to prepare a soil solution for the measurements of pH and electrical conductivity (Thermo Orion 720A, USA) (Jackson, 1962). For determination of soil humidity, soil samples were dried at 105°C for 48 h and measured for weight loss. The content of organic matter in soils was determined by measuring the loss-on ignition (Ball, 1964). In addition, total C and N were analyzed using an elemental analyzer (Heraeus Vario III-NCH, Germany). Chlorophyll-*a* (Chl-*a*) in soil samples was extracted with 90% acetone and quantified according to the methods described by Tsujimura et al. (2000).

### Statistical analysis

The indices of soil algal communities were estimated: Shannon species diversity index (H) by H=-Σ(*p*_*i*_)(log_2_*p*_*i*_), where *p*_*i*_ is the proportion of the individual species (Shannon and Weaver, 1949); evenness index (E) by E=H/H_max_, where H_max_=log_2_(S) and S the total number of species (Margalef, 1958); Simpson’s diversity index (D) by D=1-Σ(*p*_*i*_)^2^ (Simpson, 1949). These indices were calculated, using the EstimateS 8.2 software (Colwell, 2009). The data were also subjected to Pearson correlation analysis using SYSTAT (version 12, Systat Software Inc., Richmond CA, USA). Differences were considered to be significant at *p*<0.05 level.

## Results

### Physicochemical properties of soil

The studied farmlands, with exception in rice paddy, are acidified with pH ranging between 4.09 and 6.48 (Table 1). The lowest pH was measured for tea garden, while the highest for rice paddy. The acidity exhibited a close correlation with the soil content of total nitrogen (Table 2), indicating a possible result of fertilizer application in these lands.

**Table 1:** **Physicochemical properties of soils in various farmlands in mid Taiwan**

Parameter	Rice paddy	Vegetable farm	Sugarcane farm	Tea garden	Orchard
pH (in H_2_O)	7.73±0.88	6.48±0.18	6.14±0.83	4.09±0.61	5.61±0.32
EC (μS/cm)	232±65	175±36	73±16	114±28	146±29
HU (%)	13.26±3.85	7.24±2.83	2.68±1.66	4.82±2.99	7.94±3.69
Chl-*a* (μg/L)	4.69±1.20	3.14±0.86	0.88±0.28	1.81±0.57	2.93±1.01
OM (%)	3.36±0.36	2.84±0.27	1.53±0.14	2.04±0.09	2.37±0.24
TC (%)	2.91±0.24	2.77±0.20	1.88±0.13	2.18±0.18	2.56±0.22
TN (%)	0.22±0.06	0.19±0.03	0.08±0.02	0.14±0.05	0.17±0.03
Canopy coverage (%)	83.6±13.7	31.8±11.1	44.5±9.5	51.2±6.4	61.8±12.3

Chl-*a*: chlorophyll-*a*; EC: electrical conductivity; HU: humidity; OM: organic matter; TC: total carbon; TN: total nitrogen.

**Table 2:** **Pearson correlation coefficients between physicochemical variables measured at various farmlands in mid Taiwan (n=55)**

	Chl-***a***	pH	EC	HU	TC	TN	OM
pH	−0.64*						
EC	0.49	−0.68**					
HU	0.73***	−0.08	0.42				
TC	0.71**	−0.39	0.77**	0.58*			
TN	0.63*	−0.82***	0.67**	−0.11	0.73**		
OM	0.52*	−0.43	0.22	0.72*	0.83***	0.69*	
CC	0.41	−0.12	0.24	0.51	0.25	−0.11	0.39

CC: canopy coverage; Chl-*a*: chlorophyll-*a*; EC: electrical conductivity; HU: humidity; OM: organic matter; TC: total carbon; TN: total nitrogen. The significance level: ***, *p* < 0.001; **, *p* < 0.01; *, *p* < 0.05.

The highest density of soil algae (displayed as Chl-*a* content) was measured for rice paddies, where the contents of electrical conductivity (EC), humidity (HU), organic matter (OM), total carbon (TC) and total nitrogen (TN) were also high (Table 1). The density of soil algae was correlated positively with HU, TC, TN, and OM (*p*<0.05) (Table 2). A negative correlation coefficient existed between Chl-*a* content and pH values (*p*<0.05), suggesting that less soil algae were revealed in acidic lands.

### Biodiversity in different types of farmland

**Figure 1:**
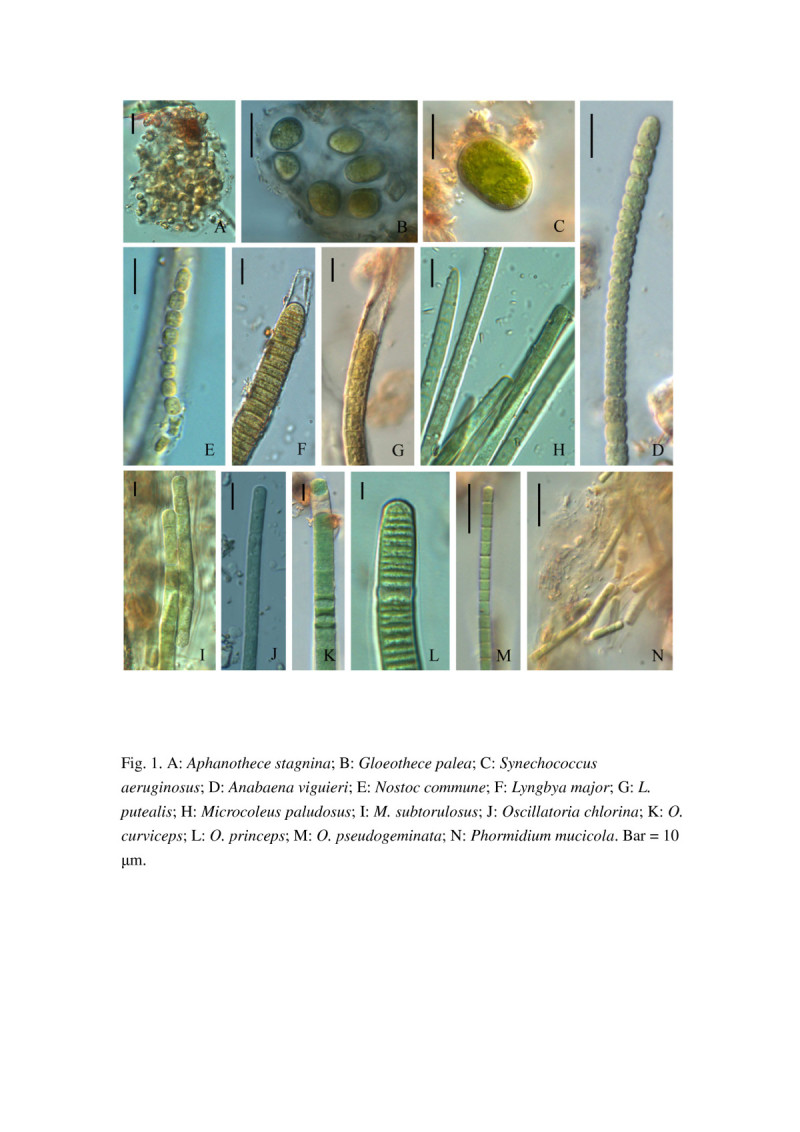
**Soil cyanobacteria found at various farmlands in mid Taiwan. A**: *Aphanothece stagnina*; **B**: *Gloeothece palea*; **C**: *Synechococcus aeruginosus*; **D**: *Anabaena viguieri*; **E**: *Nostoc commune*; **F**: *Lyngbya major*; **G**: *L. putealis*; **H**: *Microcoleus paludosus*; **I**: *M. subtorulosus*; **J**: *Oscillatoria chlorina*; **K**: *O. curviceps*; **L**: *O. princeps*; **M**: *O. pseudogeminata*; **N**: *Phormidium mucicola*. Bar = 10 μm.

**Figure 2:**
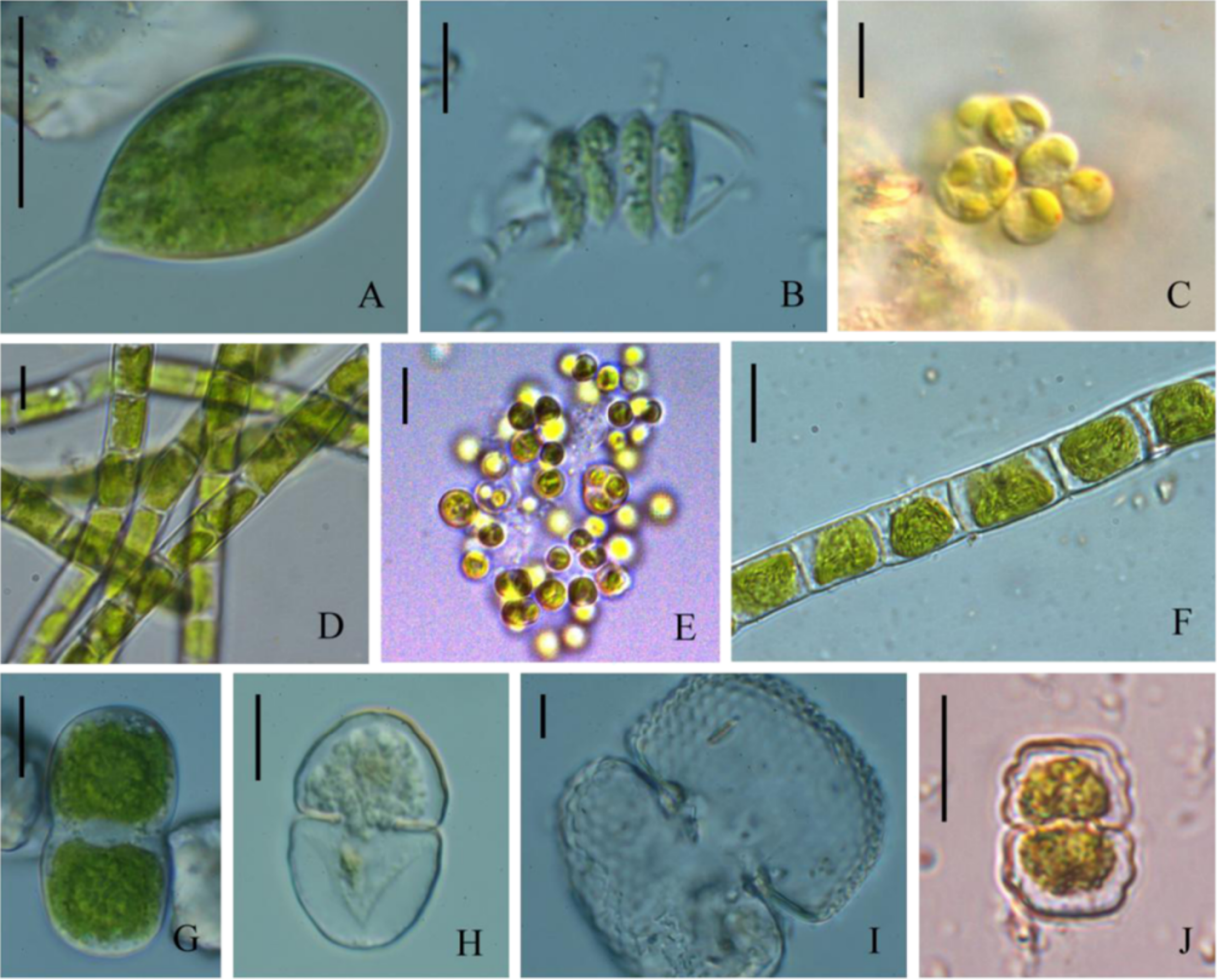
**Soil green algae found at various farmlands in mid Taiwan. A**: *Characium guttula*; **B**: *Scenedesmus quadricauda*; **C**: *Chlamydomonas reinhardtii*; **D**: *Klebsormidium flaccidum*; **E**: *Chlorella vulgaris*; **F**: *Ulothrix variabilis*; **G**: *Actinotaenium cucurbita*; **H**: *Cosmarium laeve*; **I**: *Co. quadratum*; **J**: *Co. regnellii.* Bar = 10 μm.

**Figure 3:**
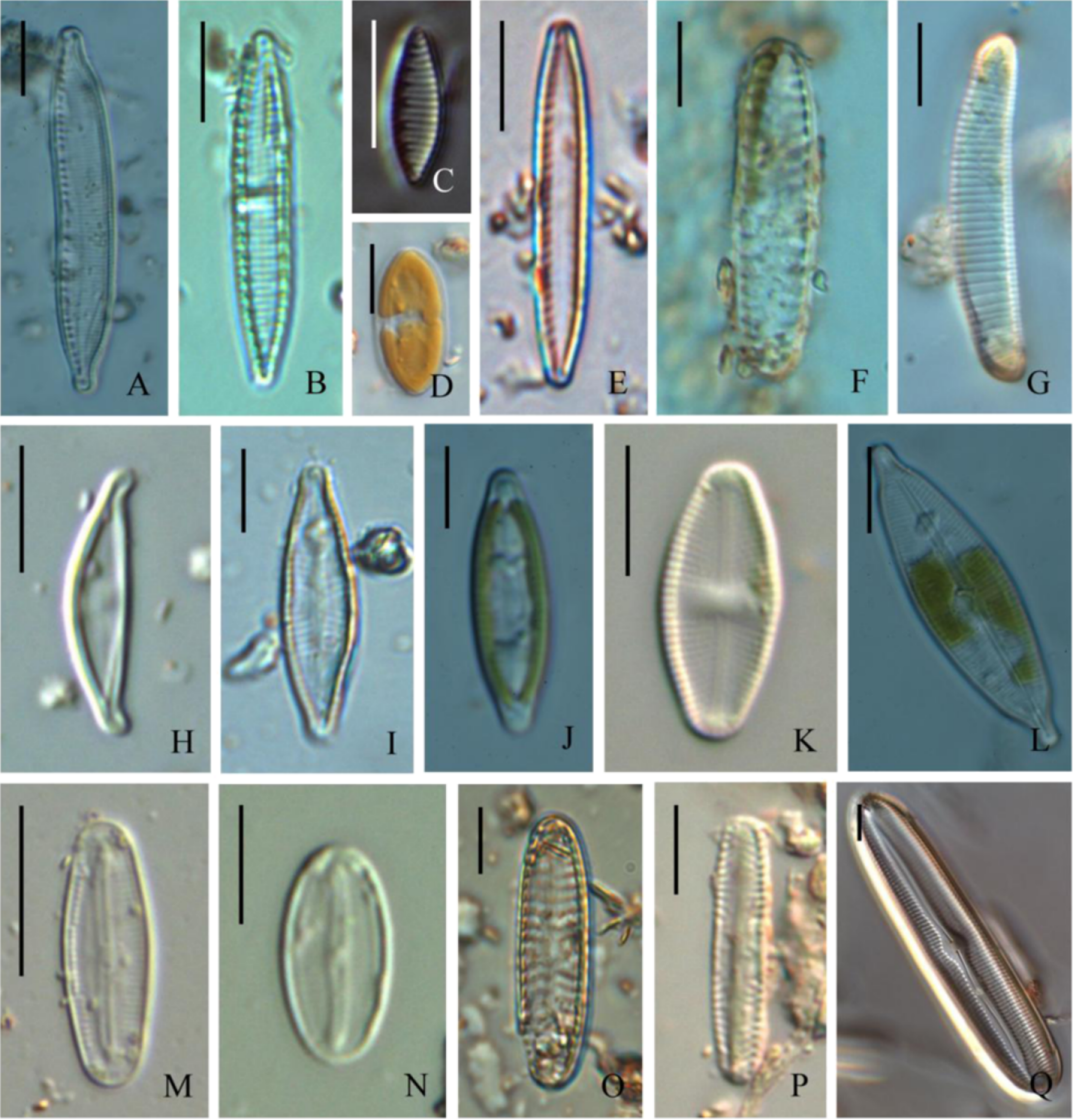
**Soil diatoms found at various farmlands in mid Taiwan. A**: *Hantzschia amphioxys*; **B**: *Nitzschia amphibian*; **C**: *Ni. amphibioides*; **D**: *Ni. levidensis*; **E**: *Ni. palea*; F: *Encyonema leei*; **G**: *Eunotia soleirolii*; **H**: *Amphora montana*; **I**: *Navicula cryptocephala*; **J**: *Na. gregaria*; **K**: *Na. mutica*; **L**: *Na. rostellata*; **M**: *Na. seminulum*; **N**: *Na. subminuscula*; **O**: *Pinnularia borealis*; **P**: *P. subcapitata*; **Q**: *P. viridis*. Bar = 10 μm.

**Figure 4:**
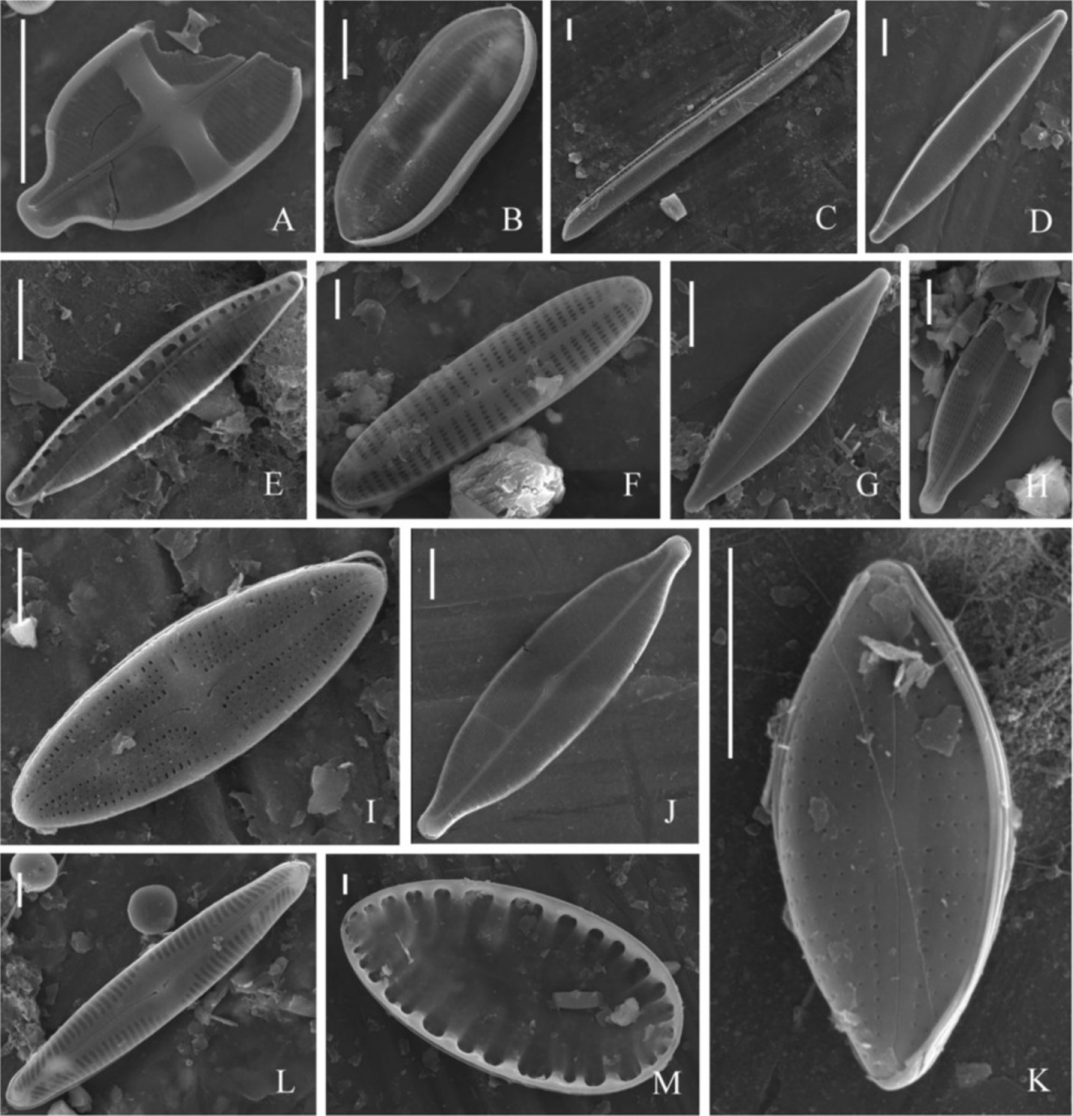
**SEM photographs of soil diatoms found at various farmlands in mid Taiwan. A**: *Achnanthes exigua*; **B**: *Nitzschia levidensis*; **C**: *Ni. obtusa*; **D**: *Ni. palea*; **E**: *Ni. pusilla*; **F**: *Encyonema leei*; **G**: *Navicula cryptocephala*; **H**: *Na. gregaria*; **I**: *Na. mutica*; **J**: *Na. rostellata*; **K**: *Na. subminuscula*; **L**: *Pinnularia gibba*; **M**: *Surirella robusta*. Bar = 5 μm.

Based on the morphology observed under the LM and SEM, a total of 64 species were identified in the studied farmlands (Table 3). They belonged to diatoms, cyanobacteria, green algae, and euglenoids in 4 classes and 33 genera. The highest diversity of species was found for the diatoms (27 species), which were mostly of the genera *Navicula*, *Nitzschia*, and *Pinnularia,* followed by cyanobacteria (22 species), and then green algae (13 species) (Tables 3 and 4). The highest number of taxa was found for the genus *Oscillatoria* (cyanobacteria). Five species are reported for the first time in Taiwan, including the cyanobacteria *M. paludosus* and *M. subtorulosus* and the diatoms *Na. subminuscula*, *Ni. levidensis*, and *Ni. pusilla* (Figures 1, 2, 3 and 4).

**Table 3:** **The checklist of abundance of soil algae found in different farmlands in mid Taiwan**

Species	Figure	Rice paddy	Vegetable farm	Sugarcane farm	Tea garden	Orchard
Cyanobacteria						
*Anabaena viguieri*	1D	C	-	-	-	-
*Anabaena* sp.		R	-	-	-	-
*Aphanothece stagnina*	1A	R	R	-	-	-
*Gloeothece palea*	1B	R	-	-	-	-
*Gloeocapsa* sp.		-	-	-	C	-
*Lyngbya major*	1 F	R	R	-	-	-
*L. putealis*	1G	R	-	-	-	-
*Lyngbya* sp.		R	R	-	-	R
*Microcystis* sp.		-	R	-	-	-
*Microcoleus paludosus*	1H	-	R	-	-	-
*M. subtorulosus*	1I	R	D	-	-	-
*Nostoc commune*	1E	C	-	-	-	-
*Nostoc* sp.		R	-	-	-	-
*Oscillatoria chlorina*	1 J	R	-	-	-	-
*O. curviceps*	1 K	R	-	-	-	R
*O. princeps*	1 L	-	R	-	-	-
*O. pseudogeminata*	1 M	R	C	-	R	R
*O. tenuis*		R	R	-	R	R
*Oscillatoria* sp.		R	-	-	-	R
*Phormidium mucicola*	1 N	C	R	-	R	R
*Phormidium* sp.		R	R	-	-	R
*Synechococcus aeruginosus*	1C	C	-	-	-	R
Chlorophytes						
*Actinotaenium cucurbita*	2G	-	-	-	-	R
*Apiocystis* sp.		-	R	-	-	-
*Characium guttula*	2A	-	-	-	-	D
*Chlamydomonas reinhardtii*	2C	R	R	-	D	R
*Chlorella vulgaris*	2E	D	C	R	C	C
*Cladophora* sp.		R	C	C	C	C
*Closterium* sp.		R	-	-	-	-
*Cosmarium laeve*	2H	R	-	-	-	-
*C. quadrum*	2I	R	-	-	-	-
*C.regnellii*	2 J	-	-	R	R	-
*Klebsormidium flaccidum*	2D	R	C	R	R	R
*Scenedesmus quadricauda*	2B	R	-	-	-	-
*Ulothrix variabilis*	2 F	-	D	C	D	R
Diatoms						
*Achnanthes exigua*	4A	R	-	-	-	-
*Amphora montana*	3H	-	-	R	R	C
*Encyonema leei*	3 F, 4 F	-	-	R	R	R
*Eunotia soleirolii*	3G	-	-	-	R	-
*Hantzschia amphioxys*	3A	D	C	R	R	C
*Navicula cryptocephala*	3I, 4G	R	R	-	-	-
*Na. gregaria*	3 J, 4H	C	R	-	-	-
*Na. mutica*	3 K, 4I	-	-	-	-	R
*Na. pelliculosa*		R	R	-	-	-
*Na. rostellata*	3 L, 4 J	R	-	-	-	-
*Na. seminulum*	3 M	-	-	-	-	D
*Na. subminuscula*	3 N, 4 K	R	-	-	-	R
*Navicula* sp.		R	R	-	-	R
*Nitzschia amphibia*	3B	-	-	D	-	R
*Ni. amphibioides*	3C	R	-	-	-	-
*Ni. levidensis*	3D, 4B	C	-	-	-	-
*Ni. obtus*	4C	R	R	-	-	R
*Ni. palea*	3E, 4D	R	R	R	R	R
*Ni. pusilla*	4E	-	-	-	-	R
*Pinnularia borealis*	3O	-	R	-	-	-
*P. gibba*	4 L	R	-	-	-	-
*P. intermedia*		-	R	-	-	-
*P. obscura*		R	-	-	-	R
*P. subcapitata*	3P	-	R	R	R	-
*P. viridis*	3Q	-	R	-	-	-
*Pinularia* sp.		-	R	-	R	-
*Surirella robusta*	4 M	R	-	-	-	-
Euglenoids						
*Euglena* sp.		R	-	-	-	-
*Lepocinclis ovum*		R	-	-	-	-

Abundance: D: Dominant (>20%); C: Common (10-20%); R: Rare (<10%); -: Not found.

**Table 4:** **Summary of the total species (genus) numbers of soil algae revealed in various farmlands in mid Taiwan**

Group	Rice paddy	Vegetable farm	Sugarcane farm	Tea garden	Orchard
Cyanobacteria	18 (9)	11 (6)	0 (0)	4 (3)	8 (4)
Chlorophytes	8 (7)	6 (6)	5 (5)	6 (6)	7 (7)
Diatoms	15 (6)	12 (4)	6 (5)	7 (6)	12 (6)
Euglenoids	2 (2)	0 (0)	0 (0)	0 (0)	0 (0)
Total	43 (24)	29 (16)	11 (10)	17 (15)	27 (17)

The soil algae in the rice paddies and vegetable farms were characterized by the dominance of cyanobacteria, a paucity of green algae, and a diverse flora of diatoms (Table 4). The dominant species in the rice paddies were *Chlorella vulgaris* and *Hantzschia amphioxys*, whereas those in the vegetable farms were *M. subtorulosus* and *Ulothrix variabilis.* The orchards were dominated by the green alga *Charactium guttula* and a diverse species of diatoms with a dominance of *Na. seminulum*. Relatively few species were found in the tea plantations, which were dominated by chlorophytes (such as *Chlamydomonas reinhardtii* and *U. variabilis*). The smallest number of species was found in the sugar cane farms, with only a few species of chlorophytes and diatoms inhabiting this habitat (Tables 3 and 4).

In studied farmlands, the community structure of soil algae varied significantly from type to type, with Shannon diversity index ranging between 1.89 and 4.63, evenness index between 0.54 and 0.93, and Simpson’s index between 0.44 and 0.91 (Table 5). The highest index values were measured for rice paddy, while the lowest for sugarcane farms. Noteworthy was that the indices in vegetable farms exhibited similar magnitude to that in orchards. The either types of lands also had more similar algal biomass (Chl-*a*) and number of species and genus (Table 4) than to other types.

**Table 5:** **Diversity indices of soil algae revealed in various farmlands in mid Taiwan**

Index	Rice paddy	Vegetable farm	Sugarcane farm	Tea garden	Orchard
Shannon diversity index	4.63±0.46	3.54±0.54	1.89±0.22	2.13±0.24	3.49±0.60
Evenness index	0.93±0.03	0.88±0.05	0.54±0.04	0.72±0.03	0.85±0.05
Simpson index	0.91±0.03	0.86±0.04	0.44±0.05	0.72±0.04	0.84±0.03

### DNA sequencing for isolate identification

Eight species of soil algae were successfully isolated and purified. The rDNA was extracted for sequencing and assessed through BLAST for sequences comparable to those published in Genbank.

The closest relative taxon for the rDNA sequences of each isolate varied between 98% and 100% (Table 6). All of the sequence similarities of the isolated algae to species in Genbank were over the threshold values (i.e., 97.5%) used to distinguish between species (Stackebrandt and Goebel, 1994).

**Table 6:** **The isolated and rDNA-identified soil algae from the farmlands in mid Taiwan**

Species	Dimension (μm) (width×length)	Accession number	Closest relative taxon	Similarity
**Cyanobacteria**				
*Microcoleus paludosus*	5.3±1.2 × 9.7±3.5	JX661567	*M. paludosus* SAG 1449-1a (EF654090)	98%
*Nostoc commune*	4.6±0.9 × 5.3±0.7	JX661565	*N. commune* CCAP 1453/24 (HE974995)	99%
*Oscillatoria princeps*	5.7±2.4 × 27.4±6.8	JX661566	*O. princeps* (AF337649)	98%
**Chlorophytes**				
*Chlamydomonas reinhardtii*	9.4±3.2 × 8.4±2.8	JX661560	*C. reinhardtii* CC-503 (FJ436973)	100%
*Chlorella vulgaris*	5.4±2.4 × 5.1±2.2	JX661561	*C. variabilis* (HQ914635)	99%
*Klebsormidium flaccidum*	8.3±2.9 × 17.5±6.1	JX661562	*K. flaccidum* (DQ629183)	100%
**Diatoms**				
*Navicula cryptocephala*	6.5±0.7 × 22.6±6.3	JX661563	*N. cryptocephala* UTEX FD109 (HQ912603)	99%
*Nitzschia palea*	3.7±1.2 × 27.3±8.9	JX661564	*N. palea* (DQ288289)	99%

Of the eight isolates studied, three were of cyanobacteria (*M. paludosus*, *N. commune*, and *O. princeps*), three of chlorophytes (*Chla. reinhardtii*, *Chlo. vulgaris*, and *Klebsormidium flaccidum*), and two of diatoms (*Na. cryptocephala* and *Ni. palea*) (Table 6). Morphologically, there were four filamentous species: *M. paludosus*, *N. commune*, *O. princeps*, and *K. flaccidum.* The other species were coccoid or long oval: *Chla. reinhardtii*, *Chlo. vulgaris*, *Na. cryptocephala*, and *Ni. palea*. These eight consensus sequences were deposited in the NCBI database under the accession numbers JX661560 - JX661567.

## Discussion

The nature of soil algal communities is the result of the complex influence of vegetation type, soil properties, and climatic conditions (Quesada et al., 1995, 1998). Our previous study indicated that water content (humidity) plays a very important role in the distribution and diversity of soil algae (Lin and Wu, 2013). This was also true in the present study, which is supported by significantly positive correlation between Chl-*a* and humidity (*p*<0.001, cf. Table 2). Of the five types of farmland studied, rice paddies are kept wet or at a high humidity throughout the rice growth cycle, and the highest species diversity (H=4.63) was observed for this type of farmland (cf. Table 5). In contrast, sugar cane farms have a relatively dry soil environment compared with other types of farmland, and the lowest diversity (H=1.89) of soil alga was observed for this type.

The occurrence of soil algae in rice paddies varies with the growth stage of the rice (Roger and Reynaud, 1976). At the early growth stage, diatoms and unicellular green algae dominate. As the biomass of soil algae increases, there is a subsequent shift to dominance by filamentous green algae and non-N-fixing cyanobacteria just before panicle initiation (Choudhary et al., 2007; Choudhary, 2009). This succession may be correlated with changes in the intensity of irradiation reaching the soil surface due to the altered canopy during the growth of the rice plant (Choudhary, 2009). Various fertilizers are also applied to rice paddies depending on the age of the rice plants, which influences the kind of nutrients available for soil algae. Thus, changes in algal diversity in rice paddies are a result of the complex effects of irradiation, nutrients, and rice age. In this type of farm lands, non-heterocystous species of cyanobacteria might become dominant when fertilizers are applied and heterocystous strains are suppressed (Jutono, 1973). In the present study, some N-fixing species, including those of *Anabaena*, *Gloeothece*, *Gloeocapsa*, and *Nostoc* were found in the farmlands, together with higher amounts of the non-N-fixing species, such as *Lyngbya*, *Microcystis*, and *Oscillatoria.* Presumably, the occurrence of N-fixing species in rice paddy could be an indication of N-deficiency, though there has had periodical application of fertilizers.

Farmlands are places of intensive anthropogenic activity, including tillage and the application of fertilizers, pesticides, and herbicides. Such activities affect the physico-chemical environment of soils and thus lower the diversity of soil algae. In the highly cultivated tea plantations, for example, a very low algal diversity (H=2.132, cf. Table 5) was observed. Tea grows mainly on mountain slopes where soil is intensively worked and pesticides and herbicide are applied to keep the tea plants healthy and free of weeds. Such activities reduce the diversity (McCann and Cullimore, 1979; Megharaj et al., 1999; Mostafa and Helling, 2002) and development of characteristic soil algae (Kuzyakhmetov, 1998; Zancan et al., 2006). More importantly, the periodic application of a variety of fertilizers to enhance productivity results in the acidification of the soil environment (with a pH as low as 3.5-4.5, cf. Table 1). Therefore, it is why pH values were significantly negative correlated with TN (resulted from organic fertilizers) (*p*<0.001) and Chl-*a* (*p*<0.05) (cf. Table 2). Apparently, the acidic environment is unfavorable for the development of biological crusts on the soil surface. Acidification is thus believed to be an important factor in lowering soil algal diversity.

The cyanobacterial species frequently occurred in the studied farmlands are of *Anabaena*, *Calothrix*, *Lyngbya*, *Microcoleus*, *Nostoc*, *Oscillatoria*, *Phormidium,* while green algae of *Chlorella*, *Cladophora*, *Klebsormidium*, and diatoms of *Hantzschia*, *Navicula*, *Nitzschia*, and *Pinnularia.* These species are cosmopolitan and widespread in various types of soils in literatures (Song et al., 2005; Begum et al., 2008; Khaybullina et al., 2010). In contrast, species of cyanobacteria such as *Cylindrospermum*, *Pseudoanabaena*, *Scytonema*, and *Tricormus* occurred only rarely*.* These species are known to be susceptible to pesticides, disturbance (such as tillage), and heavy metal pollution (Zancan et al. 2006), which are commonly applied in farmlands. It is possibly why they only rarely appeared in the farmlands studied.

Nitrogen is a limiting nutrient for crop growth in many farms. Fertilizers are commonly used in N-deficient farms (Malik et al., 2001). Algal inoculation, or so-called algalization, has been applied as an alternative to fertilizer because it exerts a positive effect on soil properties (Aiyer et al., 1972) and provides better N-availability than chemical fertilizers (Tirol et al., 1983). Previous reports have shown that the use of cyanobacteria results in a better yield and improves the N content and nitrogenase activity in rice paddies through biological N fixation (Gurung and Prasad, 2005; Tripathi et al., 2008). In addition, it increases the aggregation of soil particles, the water-holding capacity, P-availability, and the quantity of microflora (Watanabe and Roger, 1984). Cyanobacterial biofertilizers are eco-friendly and cost effective for farmlands. In this regard, the N-fixing species of cyanobacteria identified in this study are good candidates for biofertilizers in Taiwan.

The majority of soil organisms are also difficult to culture under laboratory conditions (Amann et al., 1995; Pace, 1997; Nübel et al., 2000). In this study, we only successfully isolated 8 isolates from 64 samples (ca. 12.5%). The identification of soil algae is difficult, because of insufficient available morphological data or similar morphological characteristics for the microorganisms grown in similar biotopes (Řeháková et al., 2007). In addition, the morphology of some algae might be altered under cultured conditions (Ward et al., 1992; Burja et al., 2001; Lin et al., 2012). Thus, we employ DNA sequence data in this study, as suggested by Dorador et al. (2008), in supplement to morphological characteristics. The DNA sequence data allow a comparison with already reported known species.

## Conclusions

This article reported for the first time the diversity of soil algae in five types of farmland in mid-Taiwan, which were supported by the data from polyphasic approaches including cultivation, morphological studies, and rDNA sequences. Sixty-four taxa of cyanobacteria, diatoms, green algae, and euglenoids were identified. The highest species diversity was observed in the rice paddy, while the lowest in sugar cane farms. The humidity and acidity were the most important environmental factors determined the diversity of soil algae in the farmlands studied.

## Authors’ original submitted files for images

Below are the links to the authors’ original submitted files for images. Authors’ original file for figure 1 Authors’ original file for figure 2 Authors’ original file for figure 3 Authors’ original file for figure 4

## Abbreviations

CC: Canopy coverage
Chl-a: Chlorophyll-*a*
D: Simpson’s diversity index
E: Evenness index
EC: Electrical conductivity
H: Shannon species diversity index
HU: Humidity
OM: Organic matter
TC: Total carbon
TN: Total nitrogen.
