# Supplementary figures and images for: Biodiversity of soil algae in the farmlands of mid-Taiwan
Source: Bot Stud. 2013 Sep 27;54:41. doi: 10.1186/1999-3110-54-41 (PMC5432821; doi:10.1186/1999-3110-54-41)

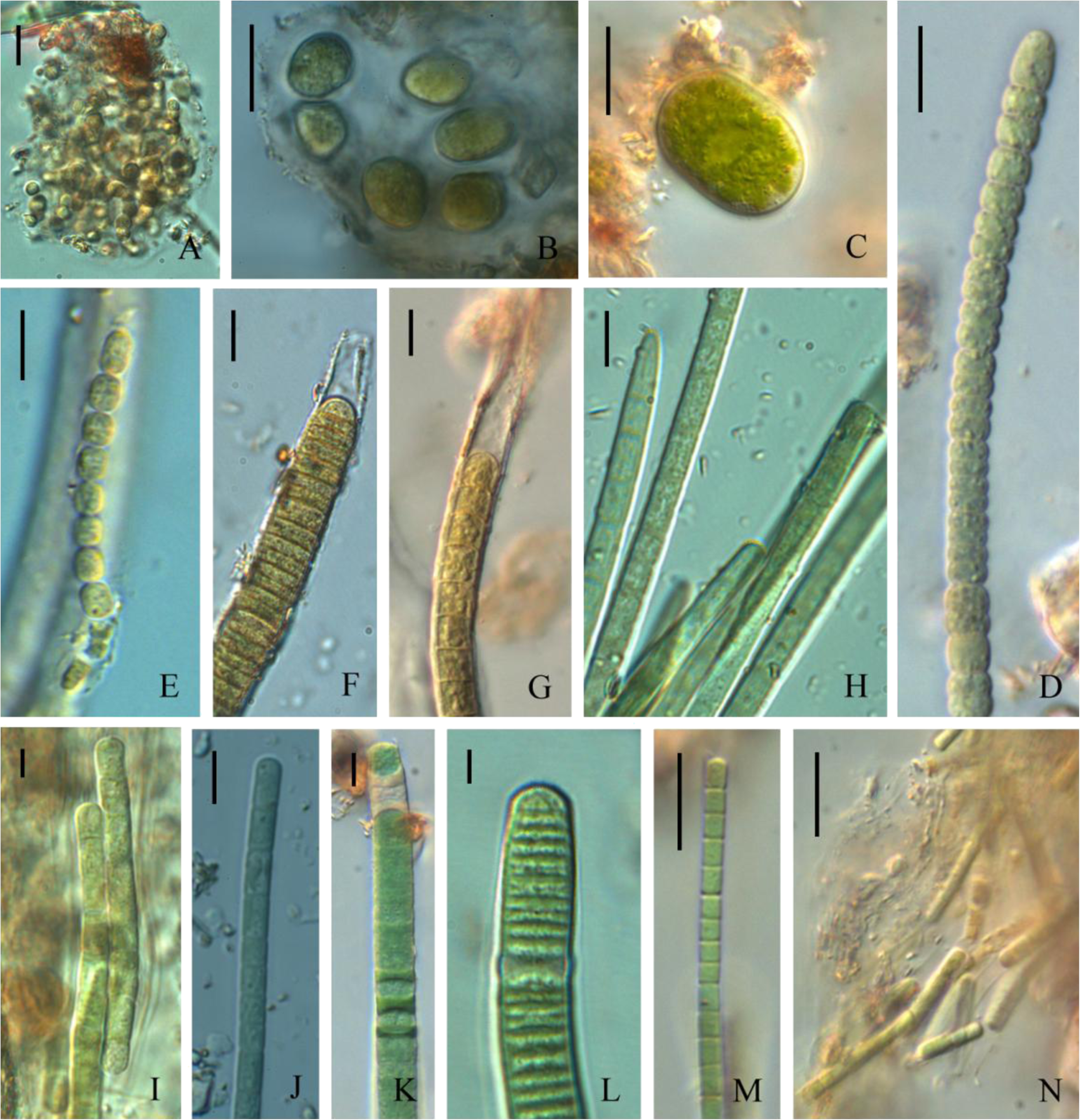

Supplement: Supplementary file 1 — Authors’ original file for figure 1 [file 40529_2013_37_MOESM1_ESM.tif]

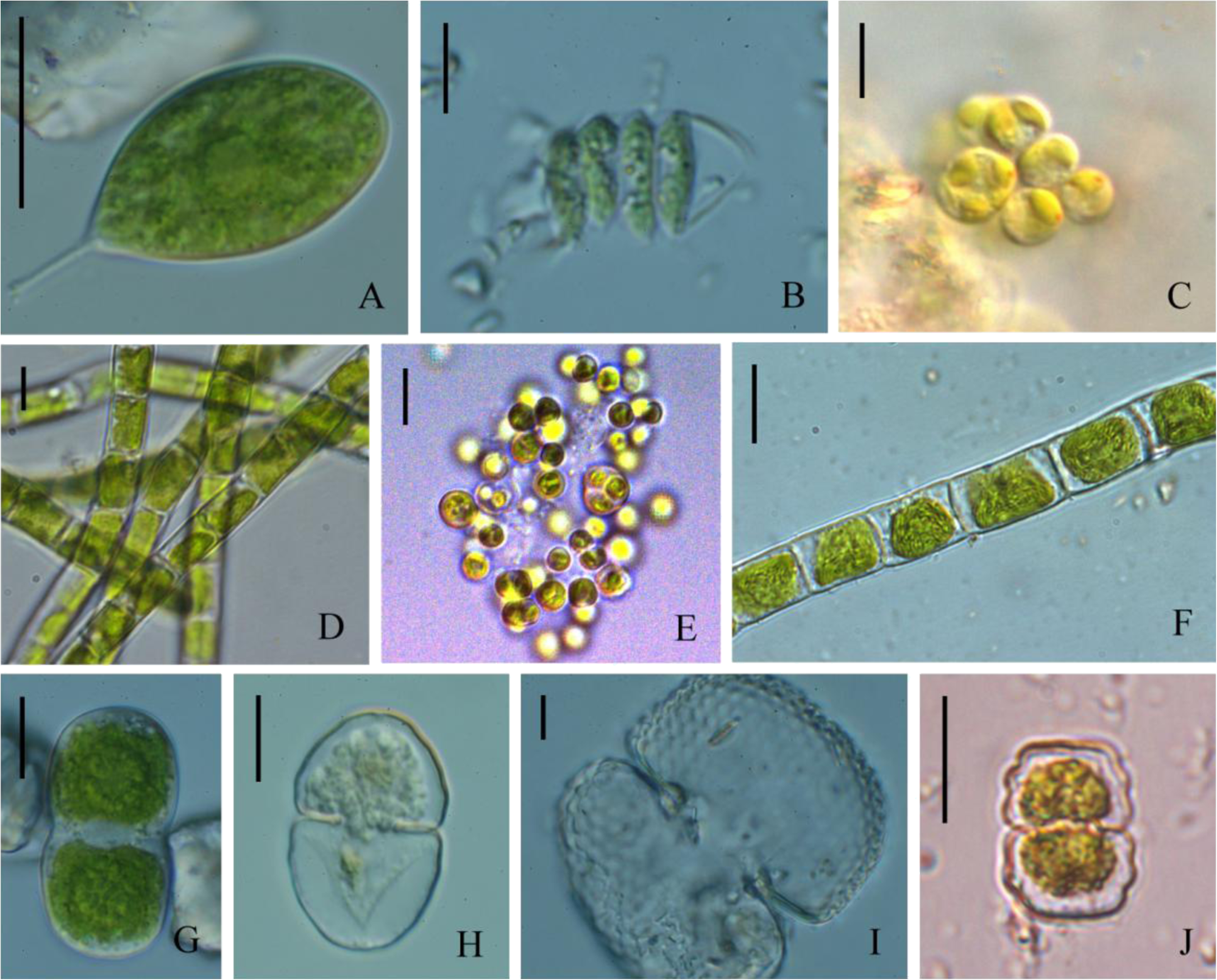

Supplement: Supplementary file 2 — Authors’ original file for figure 2 [file 40529_2013_37_MOESM2_ESM.tif]

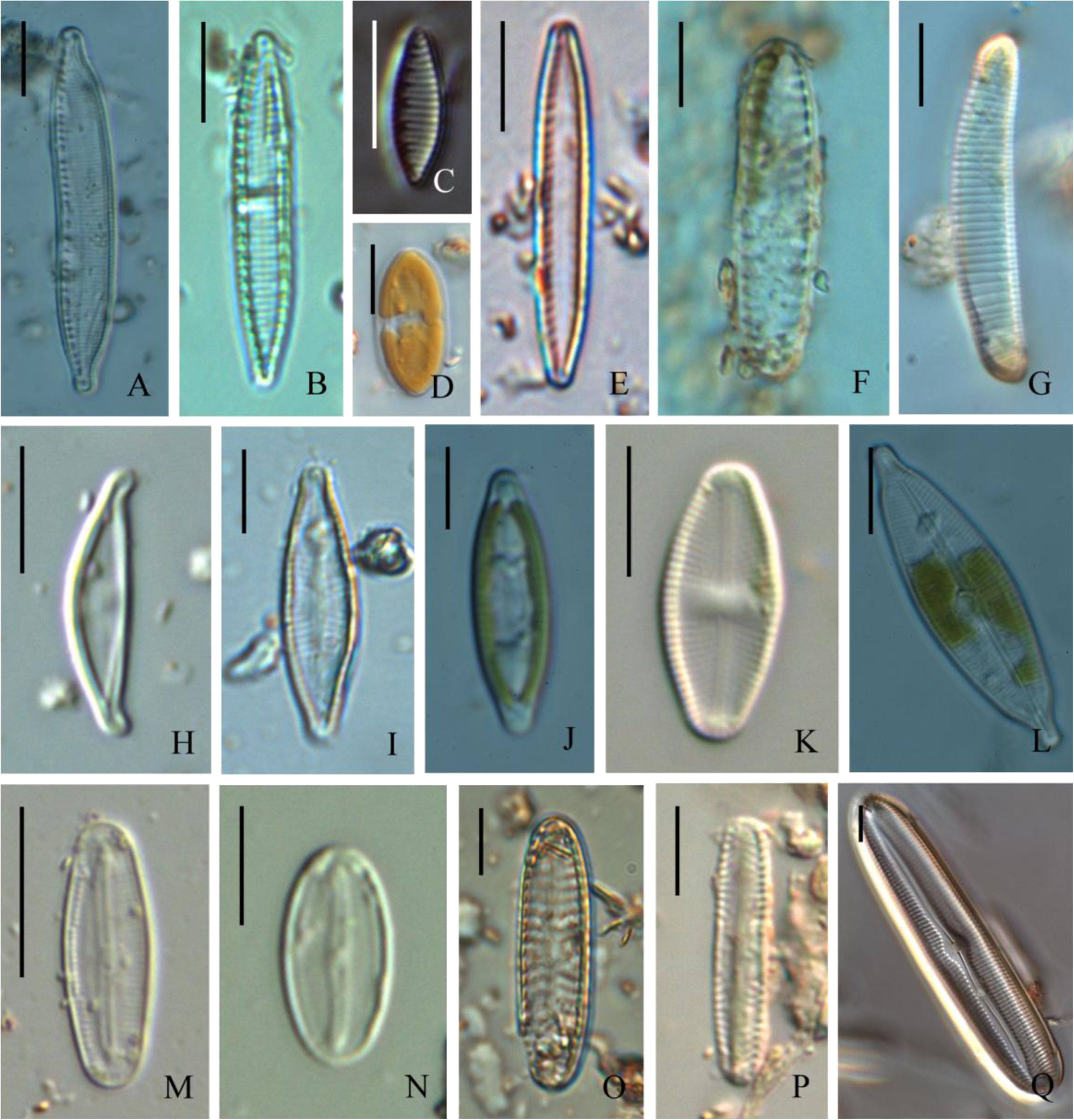

Supplement: Supplementary file 3 — Authors’ original file for figure 3 [file 40529_2013_37_MOESM3_ESM.tif]

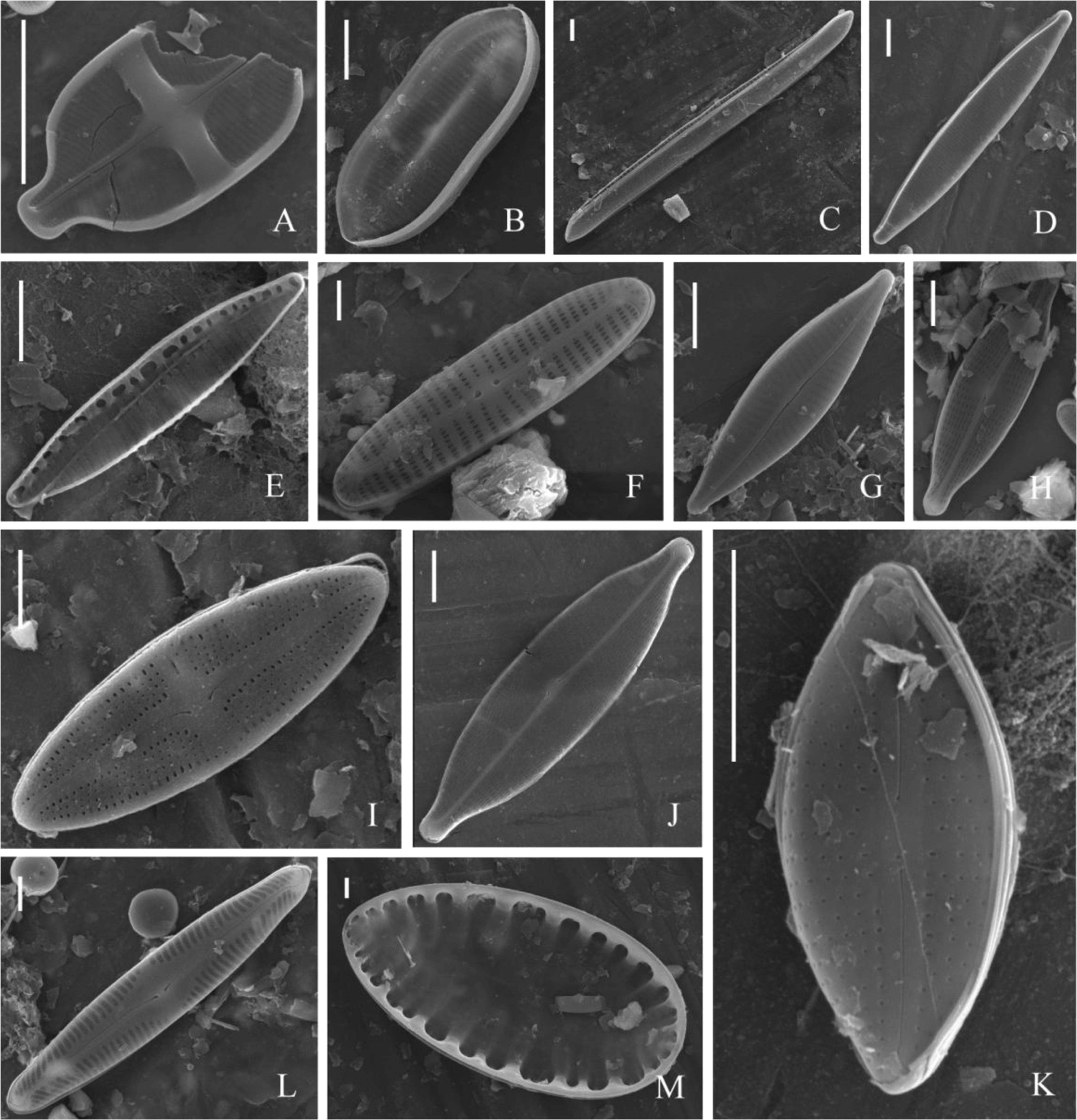

Supplement: Supplementary file 4 — Authors’ original file for figure 4 [file 40529_2013_37_MOESM4_ESM.tif]
